# Coagulation Factor Xa Has No Effects on the Expression of PAR1, PAR2, and PAR4 and No Proinflammatory Effects on HL-1 Cells

**DOI:** 10.3390/cells12242849

**Published:** 2023-12-15

**Authors:** Lukas Ruf, Alicja Bukowska, Andreas Gardemann, Andreas Goette

**Affiliations:** 1 Institute of Clinical Chemistry and Pathobiochemistry, Department of Pathobiochemistry, Otto-von-Guericke-University Magdeburg, Leipziger Str. 44, 39120 Magdeburg, Germany; lukas.ruf@med.uni-heidelberg.de (L.R.);; 2 Department of Cardiology and Intensive Care Medicine, St. Vincenz-Hospital Paderborn, Am Busdorf 2, 33098 Paderborn, Germany

**Keywords:** atrial fibrillation, atrial remodeling, atrial myocytes, HL-1 cells, FXa, FXa-induced signal transduction, protease-activated receptor, PAR1-agonist, PAR2-agonist

## Abstract

Atrial fibrillation (AF), characterised by irregular high-frequency contractions of the atria of the heart, is of increasing clinical importance. The reasons are the increasing prevalence and thromboembolic complications caused by AF. So-called atrial remodelling is characterised, among other things, by atrial dilatation and fibrotic remodelling. As a result, AF is self-sustaining and forms a procoagulant state. But hypercoagulation not only appears to be the consequence of AF. Coagulation factors can exert influence on cells via protease-activated receptors (PAR) and thereby the procoagulation state could contribute to the development and maintenance of AF. In this work, the influence of FXa on Heart Like-1 (HL-1) cells, which are murine adult atrial cardiomyocytes (immortalized), was investigated. PAR1, PAR2, and PAR4 expression was detected. After incubations with FXa (5–50 nM; 4–24 h) or PAR1- and PAR2-agonists (20 µM; 4–24 h), no changes occurred in PAR expression or in the inflammatory signalling cascade. There were no time- or concentration-dependent changes in the phosphorylation of the MAP kinases ERK1/2 or the p65 subunit of NF-κB. In addition, there was no change in the mRNA expression of the cell adhesion molecules (ICAM-1, VCAM-1, fibronectin). Thus, FXa has no direct PAR-dependent effects on HL-1 cells. Future studies should investigate the influence of FXa on human cardiomyocytes or on other cardiac cell types like fibroblasts.

## 1. Introduction

The prevalence of atrial fibrillation is estimated to be 1.5–2% in the general population in industrialised countries [1]. The prevalence and incidence of AF have been increasing in recent decades [2,3,4]. The greatest risk of AF comes from thromboembolic events. Marini et al. (2005) demonstrated electrocardiographically that 24.6% of patients with ischaemic stroke had concomitant AF [5]. Furthermore, AF was associated with an increased long-term risk of heart failure in women and men and with increased all-cause mortality compared with individuals without this cardiac arrhythmia [6]. Nearly one in three outpatients with AF had at least one hospitalisation within a year [7].

Atrial remodelling may occur as a consequence of AF or precede AF due to underlying structural heart disease [8]. Among other things, it is characterised by activation and proliferation of fibroblasts, as well as hypertrophy of cardiomyocytes and fibrosis. Furthermore, it is triggered by changes in the cellular signalling cascades [8]. Spronk et al. (2017) showed in transgenic mice with a procoagulant phenotype that hypercoagulability increased susceptibility to AF and collagen deposition in the atria. Thus, hypercoagulation appears not only to be a consequence of AF, but also to contribute to its development and maintenance [9]. In fact, coagulation factors can exert influence on cells via protease-activated receptors (PAR). Protease-activated receptors (PAR) are so-called G protein-coupled receptors (GPCR) with a heptahelical structure, whereby four isoforms are distinguished (PAR1- PAR4) [10]. Overall, the various effects of PAR activation are highly complex. The many changes caused by PAR activation primarily affect inflammatory signalling cascades [11,12]. For example, transgenic mice overexpressing PAR1 in cardiomyocytes showed eccentric hypertrophy of the heart [13].

Coagulation factor X (also called Stuart-Prower factor) connects the extrinsic and intrinsic pathways in the blood coagulation cascade. Factor Xa is a serine protease and belongs to the vitamin K-dependent coagulation factors. In recent years, there has been more evidence of PAR-mediated effects of some coagulation factors, also outside the blood clotting, on specific tissues and cells [10]. FXa can activate PAR1, PAR2, and PAR4 [14,15,16]. Thrombin, on the other hand, can activate PAR1, PAR3, and PAR4 [16,17,18]. In human right atrial tissue slices, FXa stimulated an inflammatory response via PAR1 and PAR2 [19]. However, cardiac tissue is composed of cardiomyocytes, endothelial cells, perivascular cells, and fibroblasts [20], and overall FXa-mediated PAR-dependent effects specifically on cardiomyocytes are poorly studied. There is much evidence on the effects of FXa on fibrobalsts. FXa initiated both profibrotic and proinflammatory signalling cascades in murine embryonic fibroblasts via PAR2 [21]. In contrast, FXa stimulated procollagen synthesis and extracellular matrix production via PAR1 in human and murine lung fibroblasts [22]. Guo et al. (2020) also demonstrated this preferential signalling pathway of FXa via PAR1 in cardiac neonatal rat fibroblasts. FXa led to phosphorylation of the MAP kinases ERK1/2 via PAR1, whereas PAR2 played no role in signal transduction [23]. Thus, the fibroblasts showed different FXa-mediated PAR1 or PAR2 signal transduction depending on animal species, biological age, and tissue type, among other factors. In conclusion, the origin of the fibroblasts seems to have an impact on PAR activity.

To the best of our knowledge, so far only Guo et al. (2020) demonstrated in neonatal rat ventricular cardiomyocytes that FXa-mediated activation of PAR1 and PAR2 led to an eccentric hypertrophic phenotype, increased ANP expression, and enhanced phosphorylation of ERK1/2 and ERK 5 [23]. Therefore, in this study the effects of FXa via PAR1, PAR2, and PAR4 on another cardiomyocyte cell line were investigated. The aim was to further decipher the influence of FXa on murine atrial cardiomyocytes of the HL-1 cell line.

## 2. Materials and Methods

### 2.1. Cell Culture

Cell culture was performed using HL-1 cells provided by W. C. Claycomb, Ph.D. (LSU Health Sciences Center, New Orleans, LA, USA). The optimal cell culture conditions of the HL-1 cells were a temperature of 37 °C, a CO_2_ content of 5%, and a humidity of 95% [24]. The full medium was changed daily. The dye trypan blue, which stains avital cells blue, was used to reassure the vitality of the cell culture.

After reaching confluence of the HL-1 cells in the full medium, they were washed with PBS and a 24-h serum deprivation was carried out to synchronise the cell phases by means of a deficiency medium. The deficiency medium was then replaced by a deficiency medium that had been substituted with the respective stimulant. These were FXa by Haemochrom Diagnostica, Essen, DE, or the murine PAR1 and PAR2 agonists (PAR1-AG and PAR2-AG) by Bachem, Bubendorf, CH. Possible concentration-dependent effects of FXa were investigated using the following concentrations: 5 nM, 10 nM, 20 nM, 30 nM, and 50 nM. The effects of a FXa concentration of 100 nM were also investigated molecularly. The concentrations of PAR1-AG and PAR2-AG were 20 µM each. To investigate possible time-dependent effects, different incubation times were used. These were 4 h and 24 h for FXa, PAR1-AG, and PAR2-AG in all experiments. In addition, the effects of a 15-, 30-, and 60-min incubation with FXa, PAR1-AG, and PAR2-AG were examined for ERK1/2 and NF-κB.

### 2.2. RT-PCR

The RNA isolation kit from Analytik Jena was used to isolate the RNA. To obtain cDNA, the cDNA synthesis kit “RevertAid™ First Strand” from the manufacturer Fermentas was used. In the reaction set-up, the cDNA was synthesised in Bio-Rad’s iCycler™ (München, Germany. For each sample, a reaction mixture (25 µL) composed of DEPC-treated water 9.5 µL by Roth, Karlsruhe, DE; SensiMix™ 12.5 µL by Bioline, London, GB; cDNA 0.5 µL and 5 µM Primermix 2.5 µL by Eurofine. The mouse primers used with their respective sequence (5′-3′) for the RT-PCR were (US: Upstream, DS: Downstream):

PAR1 (US: AGCCAGCCAGAATCAGAGAG; DS: TCGGAGATGAAGGGAGGAG), PAR4 (US: AGCCGAAGTCCTCAGACAAG; DS: GCAAGTGGTAAGCCAGTCGT), ICAM-1 (US: CTTCCTCATGCAAGGAGGAC; DS: CACTCTCCGGAAACGAATAC), VCAM-1 (US: GTTTGGAAGTAACCTTTACTC; DS: CCATCCTCATAGCAATTAAGG), FN (US: CTGGTGGCTACATGTTAGAG; DS: CTGCGGTTGGTAAATAGCTG).

RT-PCR was carried out in Bio-Rad’s iCycler™. Melting curve analysis allowed for the quality assessment of the PCR products. The collected data were calculated using the ∆∆Ct method from Bio-Rad.

### 2.3. Western Blot Analysis

The proteins separated by gel electrophoresis were transferred to a carrier polyvinylidene fluoride (PVDF) membrane by western blot. After the proteins had been transferred to the PVDF membrane by Western blot, the membranes were incubated with the primary antibodies (see Table 1) for 12–16 h or until the next day at 4 °C.

This was followed by incubation with the horseradish peroxidase coupled secondary antibody (Cell Signaling, Danvers, MA, USA) for 1.5 h at room temperature. For detection, the substrates luminol and hydrogen peroxide (Takara Holdings, Kyoto, Japan) were catalysed in a 1:1 ratio by the chemiluminescence reaction. The quantitative densitometric evaluation of the bands was performed with the software Image Studio Digits 5.2 (LI-COR Biosciences, Lincoln, NE, USA).

### 2.4. Statistical Analysis

The software Origin^®^ 8.5 (OriginLab Corporation, Northampton, MA, USA) was used for statistical analysis. An analysis of variance (ANOVA) was performed, where a *p*-value < 0.05 is considered statistically significant. The results are reported in this paper with the mean and standard error of the mean (mean ± SEM).

## 3. Results

### 3.1. No Change in the Expression of PAR1, PAR2, and PAR4 after FXa-Incubation

**Figure 1:**
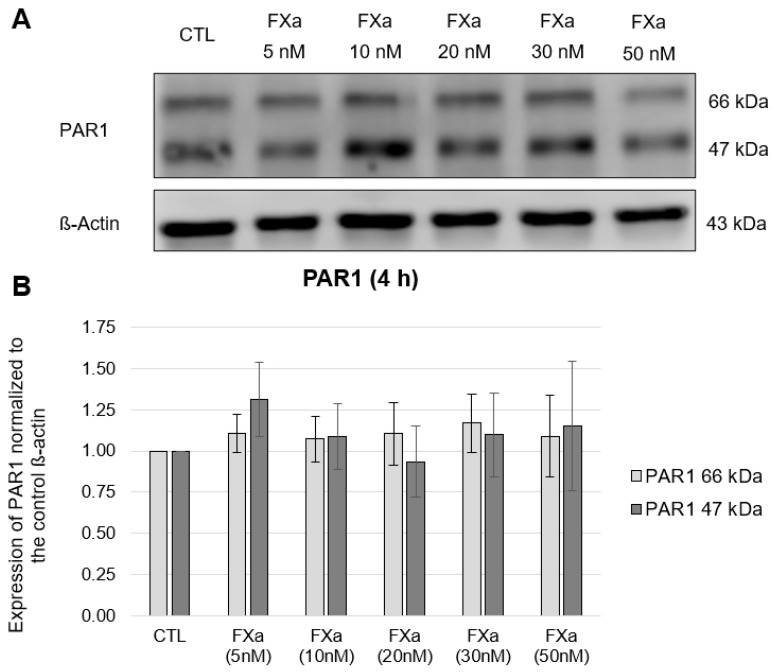
Protein expression of PAR1 in HL-1 cells after 4-h incubation with increasing concentrations of FXa (5 nM, 10 nM, 20 nM, 30 nM, 50 nM). (**A**): Representative section of a western blot. (**B**): Quantitative evaluation of PAR1 expression (mean values ± SEM) compared to the control (CTL = 1), expression of PAR1 normalized to ß-actin, n = 4.

**Figure 2:**
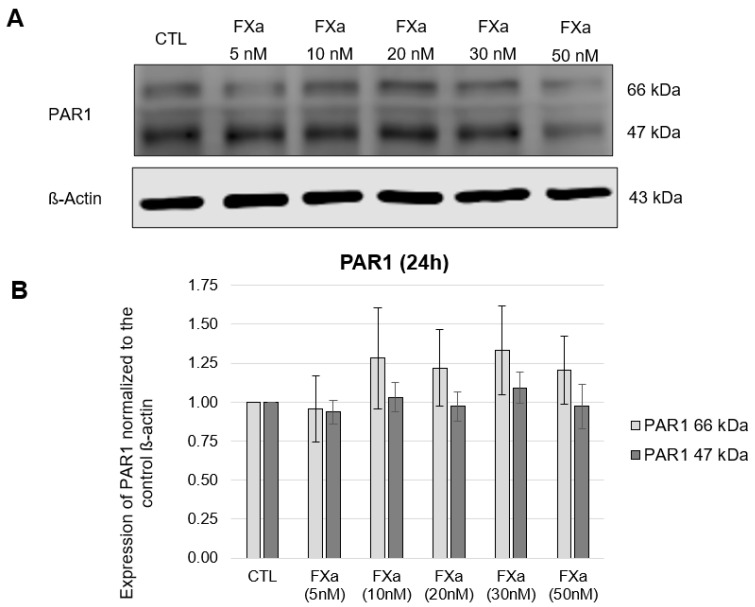
Protein expression of PAR1 in HL-1 cells after 24-h incubation with increasing concentrations of FXa (5 nM, 10 nM, 20 nM, 30 nM, 50 nM). (**A**): Representative section of a western blot. (**B**): Quantitative evaluation of PAR1 expression (mean values ± SEM) compared to the control (CTL = 1), expression of PAR1 normalized to ß-actin, n = 5.

**Figure 3:**
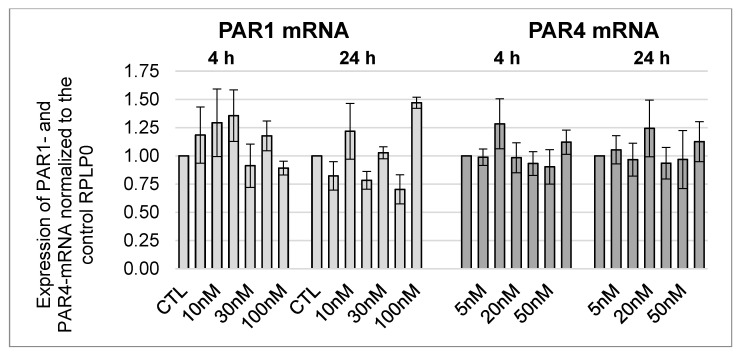
mRNA expression of PAR1 and PAR4 in HL-1 cells after 4-h and 24-h incubations with increasing concentrations of FXA (5 nM, 10 nM, 20 nM, 30 nM, 50 nM, 100 nM), mean values ± SEM compared to the control (CTL = 1). PAR1 n = 4; PAR4 n = 4.

**Figure A1:**
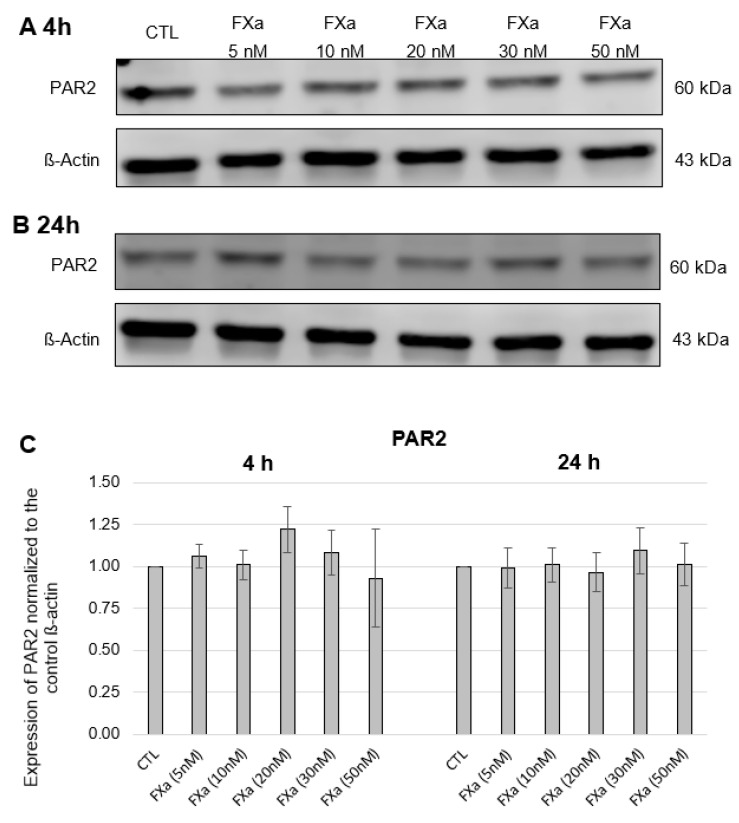
Protein expression of PAR2 in HL-1 cells after 4-h and 24-h incubations with increasing concentrations of FXa (5 nM, 10 nM, 20 nM, 30 nM, 50 nM). (**A**,**B**): Representative sections of a western blot. (**C**): Quantitative evaluation of the expression of PAR2 (mean values ± SEM) compared to the control (CTL = 1), expression of PAR2 normalized to ß-actin, n = 5.

**Figure A2:**
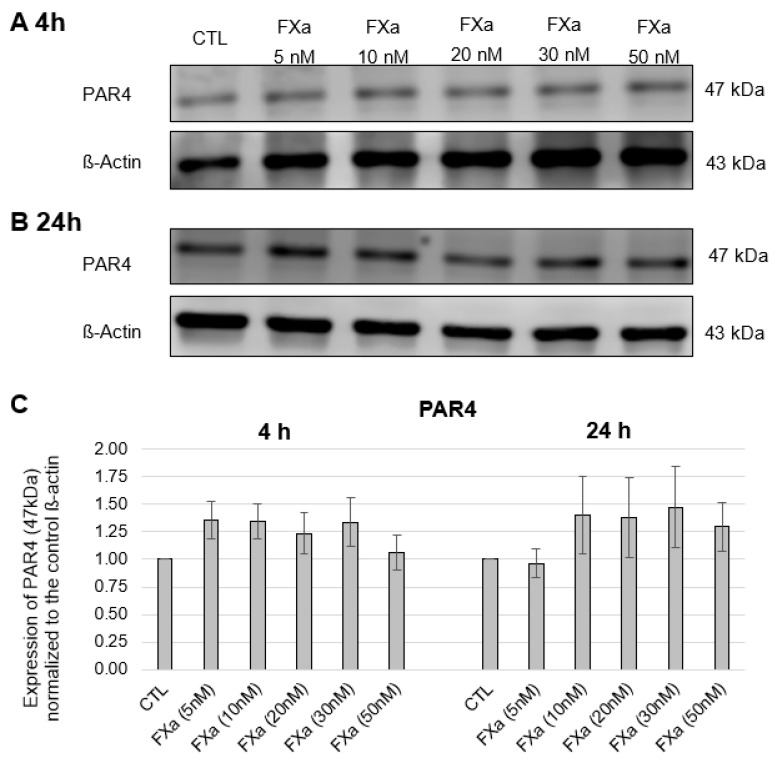
Protein expression of PAR4 in HL-1 cells after 4-h and 24-h incubations with increasing concentrations of FXa (5 nM, 10 nM, 20 nM, 30 nM, 50 nM). (**A**,**B**): Representative sections of a western blot. (**C**): Quantitative evaluation of the expression of PAR4 (mean values ± SEM) compared to the control (CTL = 1), expression of PAR4 normalized to ß-actin, n = 5.

In the HL-1 cells, both the molecular mass of 47 kDa predicted by the cDNA sequence and the post-translationally modified variant of 66 kDa resulting from N-glycosylation [25] could be detected for PAR1, without significant changes in the expression of PAR1 at the protein level (Figure 1 and Figure 2). PAR2 was detected with a molecular mass of 60 kDa and PAR4 was detected with the molecular weight of 47 kDa in the HL-1 cells, again without concentration- nor time-dependent FXa effects (data in Appendix A, Figure A1 and Figure A2). These results could also be found at the transcriptional level. In this case, the same experimental setup described above was used with the additional concentration of FXa of 100 nM. RT-PCR was used to measure the expression of PAR1 and PAR4 mRNA (Figure 3).

### 3.2. No Change in the Expression of von PAR1, PAR2 und PAR4 after PAR1- and PAR2-AG-Incubations

**Figure 4:**
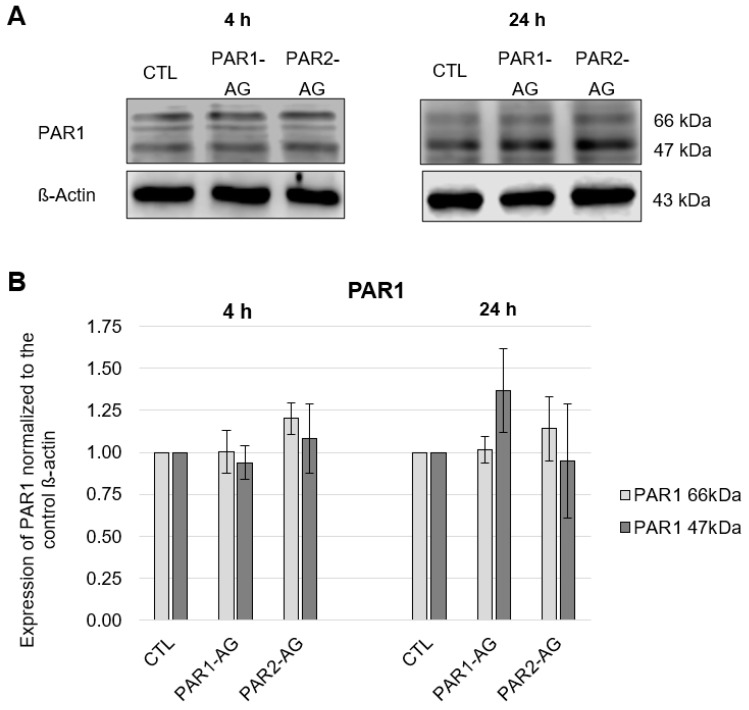
Protein expression of PAR1 in HL-1 cells after 4-h and 24-h incubations with the PAR1 and PAR2 agonists at a concentration of 20 µM each. (**A**): Representative section of a western blot. (**B**): Quantitative evaluation of PAR1 expression (mean values ± SEM) compared to the control (CTL = 1), expression of PAR1 normalized to ß-actin, n = 4.

Since FXa did not affect PAR1, PAR2, and PAR4 expressions in HL-1 cells, it was investigated whether specific PAR1 and PAR2 agonists had an effect on the protease-activated receptors in murine cardiomyocytes. The PAR agonists specifically activate their respective receptor without the need for proteolytic cleavage of the receptors and a bound ligand. As Figure 4 demonstrates, no significant changes in the glycosylated (66 kDa) and non-glycosylated (47 kDa) PAR1 form at the protein level could be detected after 4-h and 24-h incubations with PAR1 and PAR2 agonists.

**Figure 5:**
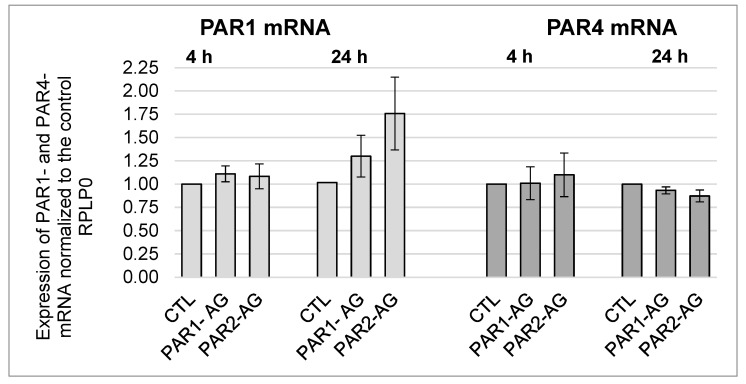
mRNA expression of PAR1 and PAR4 in HL-1 cells after 4-h and 24-h incubations with PAR1 and PAR2 agonists in a concentration of 20 µM each, with mean ± SEM compared to the control (CTL = 1). PAR1 n = 6; PAR4 n = 3.

**Figure A3:**
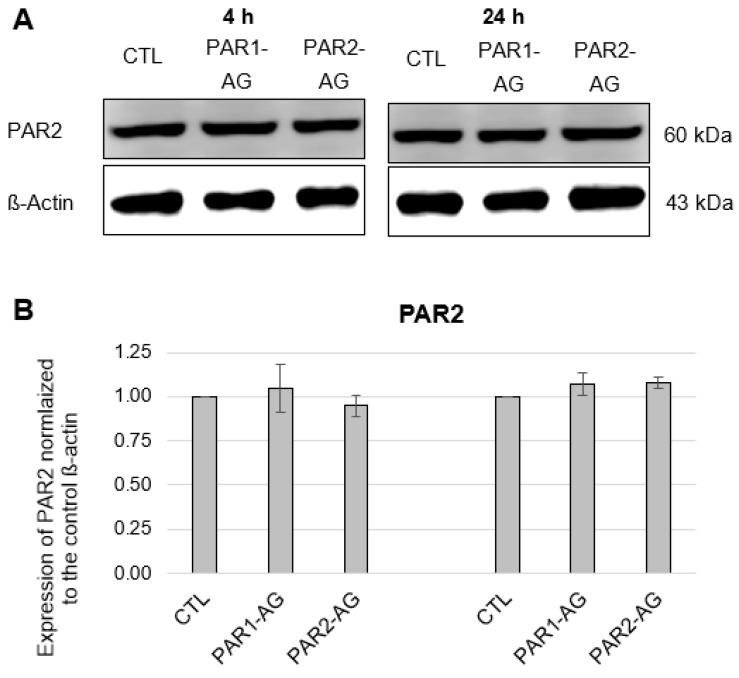
Protein expression of PAR2 in HL-1 cells after 4-h and 24-h incubations with the PAR1 and PAR2 agonists in a concentration of 20 µM each. (**A**): Representative sections of a western blot. (**B**): Quantitative evaluation of the PAR2 expression (mean values ± SEM) compared to the control (CTL = 1), expression of PAR2 normalized to ß-actin, n = 6.

**Figure A4:**
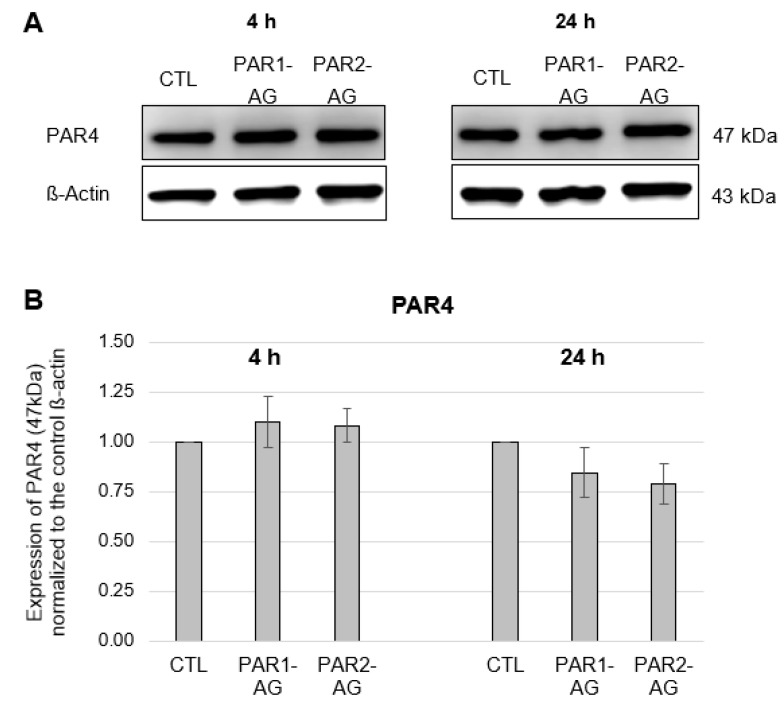
Protein expression of PAR4 in HL-1 cells after 4-h and 24-h incubations with the PAR1 and PAR2 agonists in a concentration of 20 µM each. (**A**): Representative section of a western blot. (**B**): Quantitative evaluation of the PAR4 expression (mean values ± SEM) compared to the control (CTL = 1), expression of PAR4 normalized to ß-actin, n = 6.

Furthermore, the effects of PAR1- and PAR2-agonists on the expression of PAR2 and PAR4 were analysed. No significant changes in protein expression could be detected after 4-h and 24-h incubations for PAR2 and PAR4 (data in Appendix A, Figure A3 and Figure A4). Similar investigations were carried out at the mRNA level. No significant changes in PAR1 mRNA and PAR4 mRNA were observed either (see Figure 5).

### 3.3. No Change in the Phosphorylation of ERK1/2 after FXa and PAR1- and PAR2-AG Incubations

**Figure 6:**
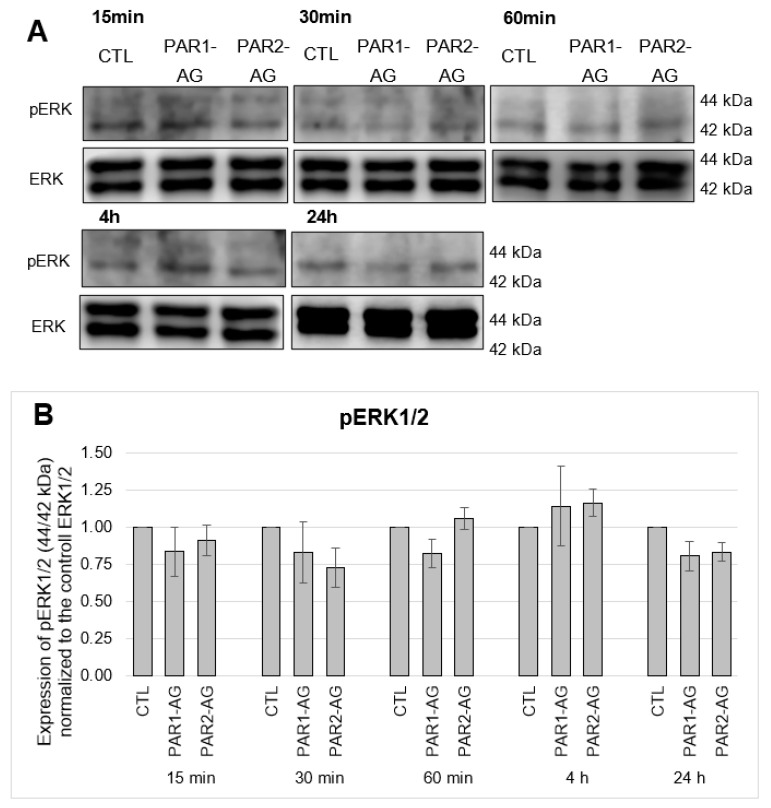
Protein expression of pERK1/2 in HL-1 cells after 15-, 30-, 60-min and 4-h and 24-h incubations with PAR1 and PAR2 agonists in a concentration of 20 µM each. (**A**): Representative section of a western blot. (**B**): Quantitative evaluation of pERK1/2 expression (mean values ± SEM) compared to the control (CTL = 1), expression of pERK1/2 (44/42 kDa) normalized to ERK1/2, n = 3.

**Figure 7:**
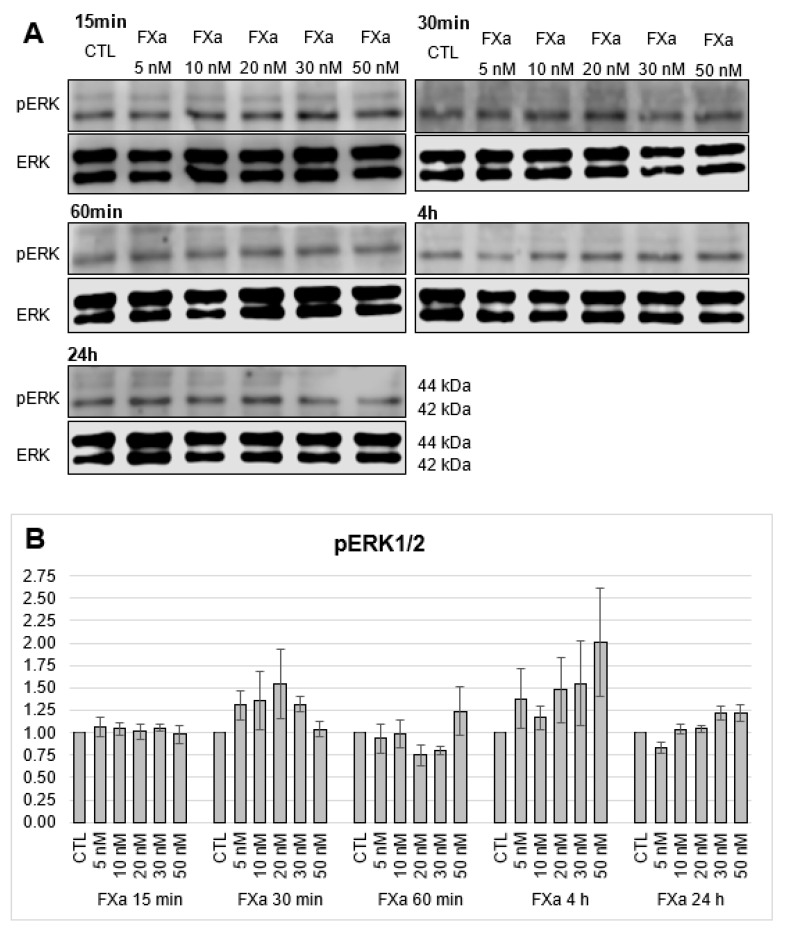
Protein expression of pERK1/2 in HL-1 cells after 15-, 30-, 60-min and 4-h and 24-h incubations with increasing concentrations of FXa (5 nM, 10 nM, 20 nM, 30 nM, 50 nM). (**A**): Representative section of a western blot. (**B**): Quantitative evaluation of pERK1/2-Expression (mean values ± SEM) compared to the control (CTL = 1), expression of pERK1/2 (44/42 kDa) normalized to ERK1/2, n = 3.

Following the cellular signalling cascade, the MAP kinase “extracellular-signal regulated kinases 1/2” ERK1/2 were tested for possible activation by phosphorylation. ERK1 is the p44 MAPK and ERK2 is the p42 MAPK [26]. Activation of PAR1 or PAR2 can lead to phosphorylation of ERK1/2 [27]. Mainly pERK2 (42 kDa) was detected while pERK1 (44 kDa) was barely expressed (Figure 6 and Figure 7). In this case, no enhanced phosphorylation of ERK1/2 was detectable upon 15-, 30-, and 60-min and 4-h and 24-h incubation with PAR1- and PAR2-agonists at the protein level (Figure 6). The possible influence of FXa on the phosphorylation of ERK1/2 was also investigated. FXa showed no activation of the MAP kinase in a concentration- and time-dependent manner (Figure 7).

### 3.4. No Change in the Phosphorylation of Transcription Factor NF-κB after FXa and PAR1- and PAR2-AG Incubations

**Figure 8:**
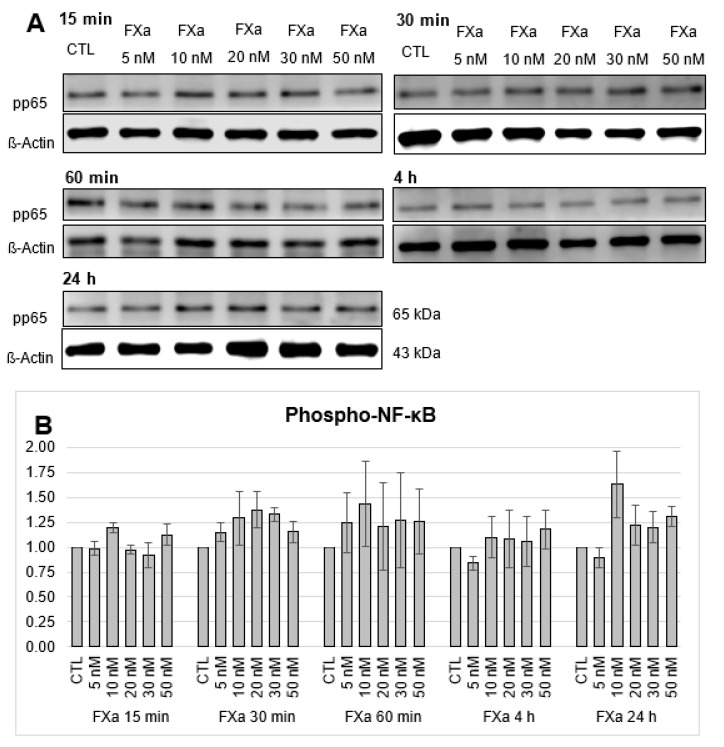
Protein expression of Phospho-NF-κB UE p65 (pp65) in HL-1 cells after 15-, 30-, 60-min and 4-h and 24-h incubations with increasing concentrations of FXa (5 nM, 10 nM, 20 nM, 30 nM, 50 nM). (**A**): Representative section of a western blot. (**B**): Quantitative evaluation of pp65 expression (mean values ± SEM) compared to the control (CTL = 1), expression of pp65 normalized to ß-actin, n = 3.

**Figure 9:**
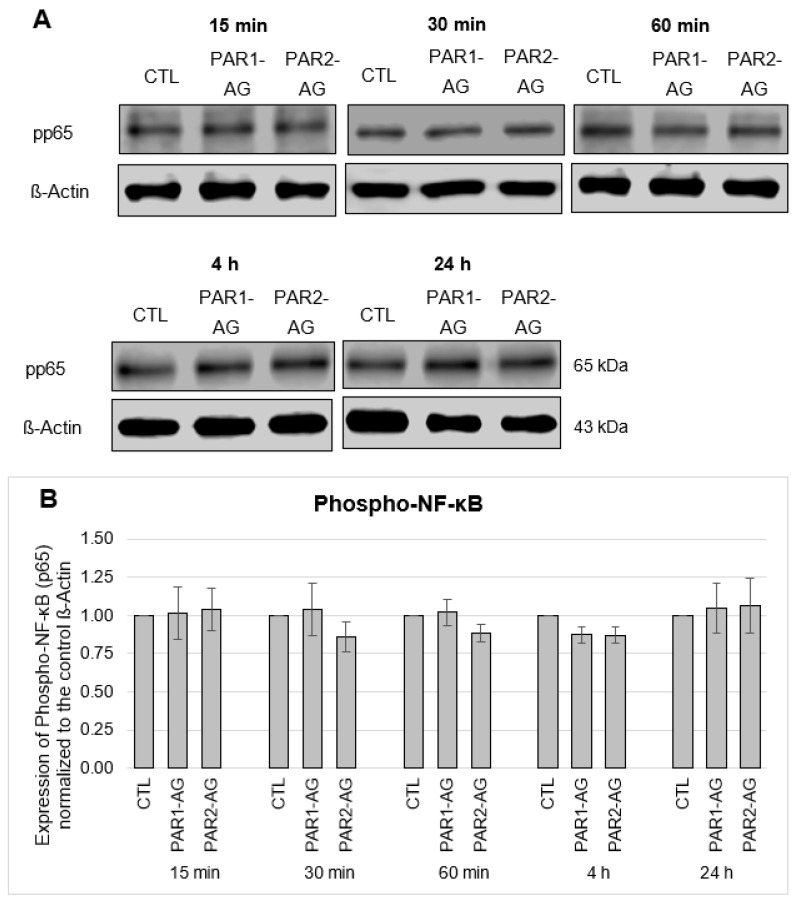
Protein expression of Phospho-NF-κB UE p65 (pp65) in HL-1 cells after 15-, 30-, 60-min and 4-h and 24-h incubations with PAR1- and PAR2- agonists in a concentration of 20 µM each. (**A**): Representative section of a western blot. (**B**): Quantitative evaluation of pp6 expression (mean values ± SEM) compared to the control (CTL = 1), expression of pp65 normalized to ß-actin, n = 5.

Next, the central transcription factor phospho-NF-κB with the subunit p65 (RelA) was examined. As Figure 8 indicates, the expression of the phosphorylated subunit p65 (pp65) of the transcription factor NF-κB did not change even with concentration- and time-dependent FXa incubations. Furthermore, no significant time-dependent effects on the phosphorylation of the p65 subunit (pp65) of the transcription factor NF-κB could be detected after incubations with the PAR1- and PAR2-agonists (Figure 9).

### 3.5. No Change in the mRNA Expression of the Cell Adhesion Molecules ICAM-1, VCAM-1, and Fibronectin after FXa and PAR1- and PAR2-AG Incubations

**Figure 10:**
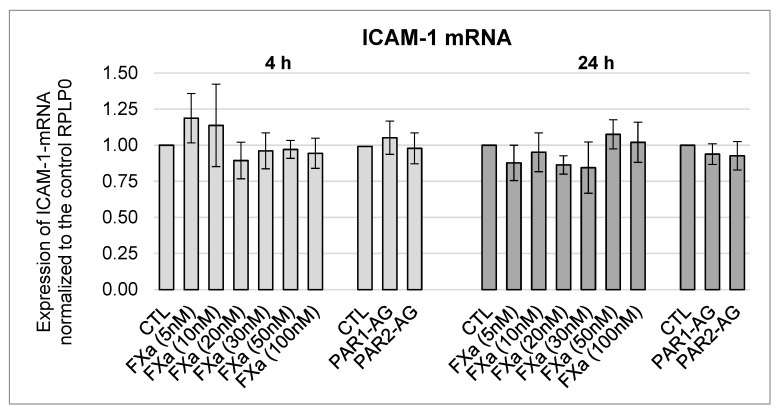
mRNA expression of ICAM-1 in HL-1 cells after 4-h and 24-h incubations with FXa in different concentrations (5 nM, 10 nM, 20 nM, 30 nM, 50 nM, 100 nM) and PAR1- and PAR2-agonists in a concentration of 20 µM each, mean ± SEM compared to the control (CTL = 1). FXa n = 4; PAR1/-2-AG n = 6.

**Figure A5:**
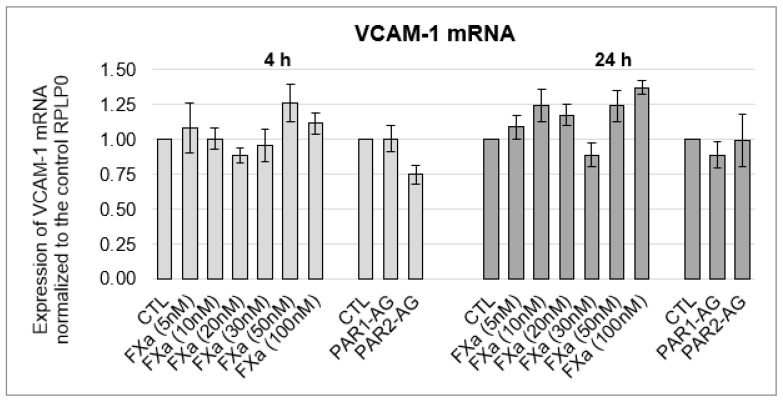
mRNA expression of VCAM-1 in HL-1 cells after 4-h and 24-h incubations with FXa in different concentrations (5 nM, 10 nM, 20 nM, 30 nM, 50 nM, 100 nM) and PAR1 and PAR2 agonists in a concentration of 20 µM each, mean ± SEM compared to the control (CTL = 1), FXa n = 4, PAR1/-2-AG n = 5.

**Figure A6:**
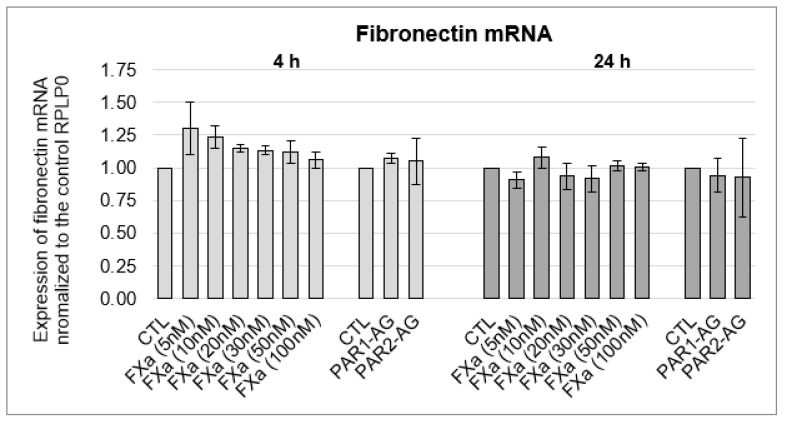
mRNA expression of fibronectin in HL-1 cells after 4-h and 24-h incubations with FXa in different concentrations (5 nM, 10 nM, 20 nM, 30 nM, 50 nM, 100 nM) and PAR1 and PAR2 agonists in a concentration of 20 µM each, mean ± SEM compared to the control CTL = 1), FXa n = 3, PAR1/-2-AG n = 3.

Possible effects of FXa on the NF-κB target genes were also investigated. The cell adhesion molecules intercellular adhesion molecule 1 (ICAM-1), vascular cell adhesion molecule 1 (VCAM-1), and fibronectin (FN) were analyzed for changes in the mRNA expression after 4-h and 24-h incubations with FXa and PAR1 and PAR2 agonists. Again, no significant time- or concentration-dependent changes were observed, as shown for ICAM-1 in Figure 10 and for VCAM-1 and FN in Appendix A Figure A5 and Figure A6.

## 4. Discussion

In this study, we have shown that FXa and murine PAR1/-2 agonists did not exert any direct effects on the cardiomyocytes of HL-1 cells via PAR.

In the present study, cardiomyocytes of the HL-1 cell line with both contractile and phenotypic adult characteristics [24,28] were shown to express PAR1, PAR2, and PAR4. In the current literature, the expression of PAR1 on cardiomyocytes has already been described, for example, in ventricular cardiomyocytes from neonatal rats [29,30]. In our study the expression of PAR1 could be shown in HL-1 cells in the non-glycosylated form (47 kDa) and the glycosylated variant (66 kDa). Interestingly, Brass et al. (1992) were able to detect only the glycosylated form (66 kDa) in HEL cells (human erythroleukemia), in platelets, and in human megakaryoblastic cells (CHRF-288) [25]. In contrast, Kuliopulos et al. were able to demonstrate a wide dispersion in the degree of glycosylation of the receptor for COS-7 fibroblasts and HEL cells, which was expressed in molecular masses of 34–100 kDa [31]. The difference in the degree of glycosylation could on the one hand be due to the fact that the different antibodies used to detect the proteins cannot recognise possible forms of N-glycosylation of PAR1. On the other hand, depending on the cell line and its origin, a different saccharide content at the N-terminal end could result from different post-translational modifications. It has been shown that a lack of N-glycosylation of PAR1 decreased cell surface expression [32,33]. The extent to which the degree of glycosylation affects the internalisation, translocation, or in particular, the function of PAR1 should be investigated in future in vitro experiments.

The non-glycosylated form of human PAR2 has the molecular weight of 36–46 kDa, while the glycosylated variant weighs 55–100 kDa. Also, a lack of N-glycosylation reduced receptor expression at the cell surface [34]. Human PAR2 has 83% of the identical amino acid sequence as murine PAR2 [35]. In this work, PAR2 protein expression in murine cardiomyocytes of the HL-1 cell line has now been demonstrated for the first time to our present knowledge. The observed molecular mass of 60 kDa could be due to deviations in the amino acid sequence or to glycosylation of PAR2 in HL-1 cells.

High PAR4 mRNA levels could be detected in human tissues via Northern blot analysis in lung, pancreas, thyroid, testis, and small intestine and lower expressions in placenta, skeletal muscle, lymph nodes, adrenal gland, prostate, uterus, and colon [36]. While no PAR4 expression has yet been detected in human heart tissue [36], weak expression was detected in murine heart tissue [17]. This was also confirmed in the cardiomyocytes of the murine HL-1 cell line, in which an expression of PAR4 with the predicted molecular mass of 47 kDa could be detected.

In the present study, no time- or concentration-dependent changes occurred in the expression of PAR1, PAR2, and PAR4 in the HL-1 cell line after FXa incubations. In contrast, Spronk et al. (2017) showed in adult atrial rat fibroblasts that thrombin increased PAR1 mRNA expression via PAR1 activation [9]. Also, FXa stimulated PAR1 mRNA expression in adult, atrial, and ventricular rat fibroblasts [37].

In our study, FXa at ascending concentrations and at different incubation times did not increase the expression of phosphorylated ERK1/2 (pERK1/2) or the phosphorylated subunit p65 (pp65) of the transcription factor NF-κB. It is proven that activation of PAR1 or PAR2 can lead to phosphorylation of ERK1/2 [27]. Increased expression of pERK1/2 has also been demonstrated in a canine model of heart failure and AF [38]. Compared to patients with sinus rhythm, patients with AF were found to have increased expression of pERK1/2 in the right atrium [39].

What are the possible reasons for the lack of stimulation by FXa in HL-1 cells? First of all, it is of great importance to characterise different cardiac models in order to gain as much knowledge as possible, especially about atrial remodelling.

Therefore, it is important to use cell cultures to generate knowledge. In order to be able to investigate a cause-effect relation between FXa and cardiomyocytes to study atrial remodelling, the HL-1 cell line was considered suitable. In contrast to our finding of no activation of PAR1 by FXa in murine adult atrial cardiomyocytes of the HL-1 cell line, Sabri et al. (2002) demonstrated a PAR1-dependent thrombin-induced increase in phosphorylation of the MAP kinases ERK1/2 in a cell culture of neonatal rat ventricular myocytes [40]. Continuing in this vein, Guo et al. (2020) showed in neonatal rat ventricular cardiomyocytes that FXa-mediated activation of PAR1 and PAR2 led to an eccentric hypertrophic phenotype, increased ANP expression. and enhanced phosphorylation of ERK1/2 and ERK 5 [23]. The differences between the cardiomyocyte cell lines could be possible reasons. The murine HL-1 cell line we investigated was shown to be suitable as an in vitro model for studying atrial remodelling, as evidenced by electrophysiological and structural changes in cell culture [41]. However, there are limitations to this model as well. HL-1 cells constantly express the SV40 large T antigen oncogene. As a result, individual HL-1 cells may be in different cell cycle phases, leading to heterogeneity in the respective cell culture [42]. Electrophysiological differences in HL-1 cell clones obtained by passengers have also been described, which may affect the comparability and reproducibility of experiments [42,43,44]. Indeed, Monge et al. (2009) also found significant differences in the energy metabolism of HL-1 cells compared to the ones in adult rat cardiomyocytes. For example, HL-1 cells showed a different spectrum of cytochromes with a sevenfold lower content of the cytochrome aa3 complex. Functionally, the respiratory chain in HL-1 cells also showed a fourfold to eightfold lower activity, measured by oxygen consumption VO2 after ADP administration. With significantly higher hexokinase activity in the HL-1 cells, the energy demand appeared to be met primarily via the glycolytic reactions [45]. Furthermore, fluorescence microscopy documented a different arrangement of mitochondria and a lack of ß-tubulin II expression in HL-1 cells compared to embryonic rat ventricular myoblasts (H9c2) [46]. Kuznetsov et al. (2015) listed, among other things, the origin of HL-1 cells from tumour cells as a possible explanation for the differences. In this context, tumour cells tend to undergo anaerobic glycolysis in the context of the so-called Warburg effect with limited mitochondrial energy production [46].

Additionally, the origin of the cell line is crucial. Depending on the species, different PAR responses can occur, which must be considered when interpreting the results. Derian et al. (1995) were able to show differences in platelet aggregation induced by PAR1 activation in different species [47]. Secondly, it is important to consider whether the cardiomyocytes originate from the atrium or the ventricle. Indeed, Nakajima et al. (2000) showed that transgenic mice with TGF-ß1 overexpressing hearts developed marked fibrosis in the atria, whereas this was not seen in the ventricles [48]. D’Alessandro et al. (2021) contrasted the cellular responses after FXa stimulation in atrial and ventricular rat fibroblasts. Firstly, the basal IL-6 mRNA expression was higher in atrial rat fibroblasts than in ventricular rat fibroblasts. Secondly, after FXa stimulation, there was an increase in IL-6 mRNA production in the ventricular rat fibroblasts, which was absent in the atrial rat fibroblasts [37]. In addition, the biological age (adult vs. neonatal) of the cardiomyocytes must be considered. It is assumed that mammalian cardiomyocytes enter the postmitotic phase within the first two neonatal weeks [49,50]. It is therefore conceivable that neonatal cardiomyocytes are more strongly influenced by external stimuli such as coagulation factors. In fact, Onódi et al. (2022) showed the differences of immortalized cell lines (HL-1, H9C2) compared to primary cardiomyocytes, e.g., in cell viability under hypoxia or in structural features. These differences illustrate the limitations of transferring the results obtained from HL-1 cells to adult human cardiomyocytes [51].

We also tested a possible PAR1 activation using the murine PAR1 agonist (PAR1-AG) and a possible PAR2 activation using the murine PAR2 agonist (PAR2-AG). In the present investigations, it was found that despite the presence of receptor expression in the cardiomyocytes of the HL-1 cell line, there was no significant activation of the receptors and the inflammatory signalling cascade we examined.

The possible influence of coagulation factors via PAR on the respective cell and tissue types depends on various factors. It could be proven that PAR are coupled and can interact with different heterotrimeric G-proteins [11,52]. Thus, depending on the corresponding intracellular equipment with the different isoforms of the G-proteins and subsequent effector proteins of the respective cells, different signalling cascades are activated and this leads to different cellular responses [11,52]. According to this, the intracellular equipment in HL-1 cells could be a cause for the lack of FXa-dependent PAR-mediated responses in HL-1 cells.

Rauch et al. (2004) showed that stimulation of human vascular smooth muscle cells (SMCs) with thrombin or FXa led to basic fibroblast growth factor (bFGF) release and autocrine bFGF-dependent PAR1 activation in terms of transactivation of the receptors (FGFR-1, PAR1) [53]. Friebel et al. (2019) has shown that patients with heart failure with preserved ejection fraction and reduced myocardial PAR2 expression have increased cardiac fibrosis. In addition, PAR2-knockout mice have increased endothelial activation, collagen deposition, and inflammation. This is due to the simultaneous reduction of the protein caveolin-1 caused by PAR2 absence, which leads to an increased expression of PAR1 and TGF-ß [54]. This shows the complex interactions of the PAR. In addition, the activation possibilities of PAR are still diverse and HL 1 cells might lack a possible coreceptor.

Furthermore, coagulation factors can also influence cells independently of PAR via other receptors or ion channels. For example, it has been shown that L-type calcium channels on cardiomyocytes are stimulated by thrombin by increasing the mean opening probability [55]. Hence, other channels or receptors on HL-1 cells might be absent or subject to different activation mechanisms, which could limit FXa signalling.

Overall, however, the evidence in the literature is mainly about FXa-mediated effects on fibroblasts. Another possible explanation for the lack of PAR activation after FXa stimulation in this work could therefore be that cardiomyocytes are not the primary target cells of coagulation factors. That is why the role of fibroblasts in cardiac remodelling is explained. In lung fibroblasts, macrophages were found to increase the expression of TGF-ß1 FXa-mediated via PAR1 [56]. Indeed, D’Alessandro et al. (2021) recently showed that FXa triggered a profibrotic and proinflammatory response via PAR1 activation in cardiac fibroblasts. Thus, increased TGF-ß1 and IL-6 mRNA expression was detected in adult rat ventricular fibroblasts and increased IL-6 mRNA expression was detected in human atrial fibroblasts following FXa stimulation [37]. Guo et al. (2020) demonstrated this preferential signalling pathway of FXa via PAR1 in cardiac neonatal rat fibroblasts: FXa led to phosphorylation of the MAP kinases ERK1/2 via PAR1, whereas PAR2 played no role in signal transduction [23]. In contrast, Bukowska et al. (2013) observed FXa-dependent PAR1- and PAR2-mediated activation of ERK1/2 and NF-κB in human atrial slices [19]. These FXa-triggered effects across different PAR isoforms could be enabled by the interactions of different cell types. Accordingly, for the human atrial tissue slices [19], the FXa responses of other cell types, such as fibroblasts, may have played a role. In addition, the fibroblasts in this cell association could be subject to other influences and hence have different FXa-dependent signal transductions.

These interactions between the different cell types in the tissue composite are obviously of particular importance. There is evidence of interactions between fibroblasts and other cells, especially in regard to possible processes in atrial remodelling. For example, Borensztajn et al. (2009) showed that FXa had no direct effects on endothelial cells in terms of cell survival or protein synthesis of fibronectin, despite triggering phosphorylation of the MAP kinase ERK1/2. After FXa stimulation of fibroblasts, their conditioned medium was added to the endothelial cells. This addition resulted in increased protein synthesis of fibronectin and proliferation and tube formation of endothelial cells. Antibodies that blocked vascular endothelial growth factor (VEGF) did not induce these effects in the endothelial cells. VEGF produced by fibroblasts after FXa stimulation was the key mediator in the conditioned medium [57]. FXa is therefore able to influence other cell types via fibroblasts. Furthermore, it has been demonstrated in the development of cardiac fibrosis that “connective tissue growth factor” (CTGF) had an autocrine profibrotic effect from fibroblasts and not from cardiomyocytes [58].

Also, there is further preliminary pathophysiological evidence of how fibroblasts may contribute to the maintenance of AF. Using the patch-clamp method, it was shown that TGF-ß1 had a direct proarrhythmogenic effect on cardiac myofibroblasts, i.e., it altered their electrophysiological phenotype [59]. Atrial dilatation also plays a role in atrial remodelling. In an in vitro model, it was shown that mechanical stretching of cardiomyocytes hardly changed excitation conduction, whereas in a cell culture of cardiomyocytes and myofibroblasts it led to a lower conduction velocity, i.e., the myofibroblasts contributed to increased voltage sensitivity and thus increased arrhythmogenicity [60]. To further understand the pathophysiology of the interplay between fibroblasts and cardiomyocytes, future studies should also investigate FXa-dependent and PAR-mediated effects on cardiac fibroblasts and uncover possible intercellular interaction pathways. In addition, the role of endothelial cells and perivascular cells in atrial remodelling should be investigated in subsequent work.

## 5. Study Limitations

Murine cells: It is essential to note the heterogeneity of PAR responsiveness in different tissues and in different species. The cardiomyocytes of the HL-1 cell line we studied might show a different PAR response compared to human cardiomyocytes due to their murine origin.

No intracellular calcium concentration measurements were performed in the present study: These could have provided further insight into possible FXa-mediated PAR-dependent effects on cardiomyocytes. For example, Jiang et al. (1998) demonstrated that high concentrations of the PAR-agonist SFLLRN, but not thrombin, increased intracellular calcium in adult rat ventricular cardiomyocytes [61]. In addition, calcium influences the inflammatory signalling cascade. For example, Macfarlane et al. (2005) showed that intracellular calcium had an influence on PAR-mediated activation of the NF-κB signalling pathway. Thus, in human dermal epithelial cells, intracellular calcium deprivation by a chelate complex resulted in decreased NF-κB DNA binding activity following PAR2 activation by trypsin [62].

Selection of FXa concentrations: For the in vitro incubations with FXa, concentrations between 5–100 nM were used, which may not correspond to the physiological FXa concentration. This was based on previous studies. Thus, the FXa concentrations used were quite similar to those of Borensztajn et al. (2008), who were able to demonstrate in vitro concentration-dependent effects of FXa on fibroblasts via PAR2 of 0.25 U/mL (=43.5 nM) to 1 U/mL (=174 nM) [21]. The concentration of factor X in human plasma is 170 nM [63,64]. To date, however, there are no data of the in vivo concentrations of the activated serine protease factor Xa. It has been shown that in vitro less than 1% of the plasma concentration of FX is required as FXa for maximal thrombin generation [64]. This low required FXa concentration in the coagulation cascade does not appear to be consistent with the concentrations for the FXa-dependent PAR-mediated effects at the cellular level. According to this, the interactions of the serine proteases with the PAR must also be considered. For example, Riewald et al. (2001) described the differences between thrombin and FXa. The binding region exosite 1 of thrombin is a basic one and that of FXa is an acidic one. This allows thrombin to bind directly to PAR and therefore has fast kinetics in receptor cleavage and thus in signal transduction as well as gene transcription. In contrast, FXa cannot dock directly to PAR and must at first bind to the cell membrane via the gamma-carboxyglutamic acid (Gla) domain [65,66]. Consequently, gene transcription through PAR activation presumably requires a relatively high FXa concentration of 10–100 nM, which would correspond to the membrane binding affinity of FXa [65].

Selection of FXa incubation times: Furthermore, the selected FXa incubation times (up to max. 24 h) might have been too short. Previous in vitro studies were also used as a guide. FXa incubation times of 2 h and 24 h on human atrial slices were sufficient to demonstrate enhanced phosphorylation of ERK1/2 and NF-κB [19]. In contrast, Guo et al. (2020) used FXa incubation times of 48 h on neonatal rat ventricular cardiomyocytes to demonstrate enhanced phosphorylation of ERK1/2 and ERK 5 [23]. In HL-1 cells, electrophysiological and structural changes in the sense of AF-induced remodelling were detected at 3.1 ± 1.3 and 9.7 ± 0.5 days after initiation of AF [41].

## 6. Conclusions

The aim of this study was to gain further insight into the cardiac remodelling processes by FXa. Therefore, the influence of FXa on HL-1 cells, which are murine adult atrial cardiomyocytes (immortalized), was investigated. PAR1, PAR2, and PAR4 expression was detected in the cardiomyocytes of the HL-1 cell line. After incubation with FXa (5 nM–100 nM; 15 min–24 h) or PAR1-/2 agonists (20 µM; 15 min–24 h), no changes occurred in PAR1, PAR2, and PAR4 expression or in the intracellular inflammatory signalling cascade. There were no time- or concentration-dependent changes in the phosphorylation of the MAP kinases ERK1/2 or the p65 subunit of NF-κB and in the mRNA expression of the cell adhesion molecules ICAM-1, VCAM-1, and fibronectin. FXa thus has no direct PAR-dependent influence on HL 1 cells. Guo et al. (2020) showed in neonatal rat ventricular cardiomyocytes that FXa-mediated activation of PAR1 and PAR2 led to an eccentric hypertrophic phenotype, increased ANP expression, and enhanced phosphorylation of ERK1/2 and ERK 5 [23]. Even though it has been proven that immortalized cell cultures like the HL-1 cells are less similar to adult cardiomyocytes and thus show lower reliability than primary cardiomyocytes [51], to the best of our knowledge, this study presented for the first time a lack of FXa effects on cardiomyocytes of atrial origin. This could be relevant in the context of the atrial remodelling by AF. Future studies should therefore investigate possible effects of FXa on human cardiomyocytes or on other cardiac cell types like fibroblasts.

## Figures and Tables

**Table 1:** Primary antibodies.

Primary Antibodies	Species	Type	Manufacturer	Dilution
Phospho- Erk 1/2	mouse	monoclonal	Cell Signaling, Danvers, MA, USA	1:500
Phospho-NF-KB p65	rabbit	monoclonal		1:500
ERK 1/2	rabbit	polyclonal		1:1000
PAR1	mouse	monoclonal	Santa Cruz, Dallas, TX, USA	1:500
PAR2	mouse	monoclonal		1:500
PAR4	mouse	monoclonal		1:500
ß-Actin	mouse	monoclonal		1:5000

## Data Availability

Data are contained within the article.

## References

[1] Camm A.J., Lip G.Y., De Caterina R., Savelieva I., Atar D., Hohnloser S.H., Hindricks G., Kirchhof P., ESC Committee for Practice Guidelines (CPG) (2012). 2012 Focused Update of the Esc Guidelines for the Management of Atrial Fibrillation: An Update of the 2010 Esc Guidelines for the Management of Atrial Fibrillation. Developed with the Special Contribution of the European Heart Rhythm Association. Eur. Heart J..

[2] Chugh S.S., Havmoeller R., Narayanan K., Singh D., Rienstra M., Benjamin E.J., Gillum R.F., Kim Y.H., McAnulty J.H., Zheng Z.J. (2014). Worldwide Epidemiology of Atrial Fibrillation: A Global Burden of Disease 2010 Study. Circulation.

[3] Miyasaka Y., Barnes M.E., Gersh B.J., Cha S.S., Bailey K.R., Abhayaratna W.P., Seward J.B., Tsang T.S. (2006). Secular Trends in Incidence of Atrial Fibrillation in Olmsted County, Minnesota, 1980 to 2000, and Implications on the Projections for Future Prevalence. Circulation.

[4] Wolf P.A., Benjamin E.J., Belanger A.J., Kannel W.B., Levy D., D’Agostino R.B. (1996). Secular Trends in the Prevalence of Atrial Fibrillation: The Framingham Study. Am. Heart J..

[5] Marini C., De Santis F., Sacco S., Russo T., Olivieri L., Totaro R., Carolei A. (2005). Contribution of Atrial Fibrillation to Incidence and Outcome of Ischemic Stroke: Results from a Population-Based Study. Stroke.

[6] Stewart S., Hart C.L., Hole D.J., McMurray J.J. (2002). A Population-Based Study of the Long-Term Risks Associated with Atrial Fibrillation: 20-Year Follow-up of the Renfrew/Paisley Study. Am. J. Med..

[7] Steinberg B.A., Kim S., Fonarow G.C., Thomas L., Ansell J., Kowey P.R., Mahaffey K.W., Gersh B.J., Hylek E., Naccarelli G. (2014). Drivers of Hospitalization for Patients with Atrial Fibrillation: Results from the Outcomes Registry for Better Informed Treatment of Atrial Fibrillation (Orbit-Af). Am. Heart J..

[8] Schotten U., Verheule S., Kirchhof P., Goette A. (2011). Pathophysiological Mechanisms of Atrial Fibrillation: A Translational Appraisal. Physiol. Rev..

[9] Spronk H.M., De Jong A.M., Verheule S., De Boer H.C., Maass A.H., Lau D.H., Rienstra M., van Hunnik A., Kuiper M., Lumeij S. (2017). Hypercoagulability Causes Atrial Fibrosis and Promotes Atrial Fibrillation. Eur. Heart J..

[10] Steinberg S.F. (2005). The Cardiovascular Actions of Protease-Activated Receptors. Mol. Pharmacol..

[11] Macfarlane S.R., Seatter M.J., Kanke T., Hunter G.D., Plevin R. (2001). Proteinase-Activated Receptors. Pharmacol. Rev..

[12] Heuberger D.M., Schuepbach R.A. (2019). Protease-Activated Receptors (Pars): Mechanisms of Action and Potential Therapeutic Modulators in Par-Driven Inflammatory Diseases. Thromb. J..

[13] Pawlinski R., Tencati M., Hampton C.R., Shishido T., Bullard T.A., Casey L.M., Andrade-Gordon P., Kotzsch M., Spring D., Luther T. (2007). Protease-Activated Receptor-1 Contributes to Cardiac Remodeling and Hypertrophy. Circulation.

[14] Camerer E., Huang W., Coughlin S.R. (2000). Tissue Factor- and Factor X-Dependent Activation of Protease-Activated Receptor 2 by Factor Viia. Proc. Natl. Acad. Sci. USA.

[15] Nystedt S., Emilsson K., Wahlestedt C., Sundelin J. (1994). Molecular Cloning of a Potential Proteinase Activated Receptor. Proc. Natl. Acad. Sci. USA.

[16] Sidhu T.S., French S.L., Hamilton J.R. (2014). Differential Signaling by Protease-Activated Receptors: Implications for Therapeutic Targeting. Int. J. Mol. Sci..

[17] Kahn M.L., Zheng Y.W., Huang W., Bigornia V., Zeng D., Moff S., Farese R.V., Tam C., Coughlin S.R. (1998). A Dual Thrombin Receptor System for Platelet Activation. Nature.

[18] Kahn M.L., Nakanishi-Matsui M., Shapiro M.J., Ishihara H., Coughlin S.R. (1999). Protease-Activated Receptors 1 and 4 Mediate Activation of Human Platelets by Thrombin. J. Clin. Investig..

[19] Bukowska A., Zacharias I., Weinert S., Skopp K., Hartmann C., Huth C., Goette A. (2013). Coagulation Factor Xa Induces an Inflammatory Signalling by Activation of Protease-Activated Receptors in Human Atrial Tissue. Eur. J. Pharmacol..

[20] Zhou P., Pu W.T. (2016). Recounting Cardiac Cellular Composition. Circ. Res..

[21] Borensztajn K., Stiekema J., Nijmeijer S., Reitsma P.H., Peppelenbosch M.P., Spek C.A. (2008). Factor Xa Stimulates Proinflammatory and Profibrotic Responses in Fibroblasts Via Protease-Activated Receptor-2 Activation. Am. J. Pathol..

[22] Blanc-Brude O.P., Archer F., Leoni P., Derian C., Bolsover S., Laurent G.J., Chambers R.C. (2005). Factor Xa Stimulates Fibroblast Procollagen Production, Proliferation, and Calcium Signaling Via Par1 Activation. Exp. Cell Res..

[23] Guo X., Kolpakov M.A., Hooshdaran B., Schappell W., Wang T., Eguchi S., Elliott K.J., Tilley D.G., Rao A.K., Andrade-Gordon P. (2020). Cardiac Expression of Factor X Mediates Cardiac Hypertrophy and Fibrosis in Pressure Overload. JACC Basic Transl. Sci..

[24] Claycomb W.C., Lanson N.A., Stallworth B.S., Egeland D.B., Delcarpio J.B., Bahinski A., Izzo N.J. (1998). Hl-1 Cells: A Cardiac Muscle Cell Line That Contracts and Retains Phenotypic Characteristics of the Adult Cardiomyocyte. Proc. Natl. Acad. Sci. USA.

[25] Brass L.F., Vassallo R.R., Belmonte E., Ahuja M., Cichowski K., Hoxie J.A. (1992). Structure and Function of the Human Platelet Thrombin Receptor. Studies Using Monoclonal Antibodies Directed against a Defined Domain within the Receptor N Terminus. J. Biol. Chem..

[26] Pearson G., Robinson F., Beers Gibson T., Xu B.E., Karandikar M., Berman K., Cobb M.H. (2001). Mitogen-Activated Protein (Map) Kinase Pathways: Regulation and Physiological Functions. Endocr. Rev..

[27] Ossovskaya V.S., Bunnett N.W. (2004). Protease-Activated Receptors: Contribution to Physiology and Disease. Physiol. Rev..

[28] Field L.J. (1988). Atrial Natriuretic Factor-Sv40 T Antigen Transgenes Produce Tumors and Cardiac Arrhythmias in Mice. Science.

[29] Jiang T., Kuznetsov V., Pak E., Zhang H., Robinson R.B., Steinberg S.F. (1996). Thrombin Receptor Actions in Neonatal Rat Ventricular Myocytes. Circ. Res..

[30] Glembotski C.C., Irons C.E., Krown K.A., Murray S.F., Sprenkle A.B., Sei C.A. (1993). Myocardial Alpha-Thrombin Receptor Activation Induces Hypertrophy and Increases Atrial Natriuretic Factor Gene Expression. J. Biol. Chem..

[31] Kuliopulos A., Covic L., Seeley S.K., Sheridan P.J., Helin J., Costello C.E. (1999). Plasmin Desensitization of the Par1 Thrombin Receptor: Kinetics, Sites of Truncation, and Implications for Thrombolytic Therapy. Biochemistry.

[32] Tordai A., Brass L.F., Gelfand E.W. (1995). Tunicamycin Inhibits the Expression of Functional Thrombin Receptors on Human T-Lymphoblastoid Cells. Biochem. Biophys. Res. Commun..

[33] Soto A.G., Trejo J. (2010). N-Linked Glycosylation of Protease-Activated Receptor-1 Second Extracellular Loop: A Critical Determinant for Ligand-Induced Receptor Activation and Internalization. J. Biol. Chem..

[34] Compton S.J., Sandhu S., Wijesuriya S.J., Hollenberg M.D. (2002). Glycosylation of Human Proteinase-Activated Receptor-2 (Hpar2): Role in Cell Surface Expression and Signalling. Biochem. J..

[35] Bohm S.K., Kong W., Bromme D., Smeekens S.P., Anderson D.C., Connolly A., Kahn M., Nelken N.A., Coughlin S.R., Payan D.G. (1996). Molecular Cloning, Expression and Potential Functions of the Human Proteinase-Activated Receptor-2. Biochem. J..

[36] Xu W.F., Andersen H., Whitmore T.E., Presnell S.R., Yee D.P., Ching A., Gilbert T., Davie E.W., Foster D.C. (1998). Cloning and Characterization of Human Protease-Activated Receptor 4. Proc. Natl. Acad. Sci. USA.

[37] D’Alessandro E., Scaf B., Munts C., van Hunnik A., Trevelyan C.J., Verheule S., Spronk H.M.H., Turner N.A., Ten Cate H., Schotten U. (2021). Coagulation Factor Xa Induces Proinflammatory Responses in Cardiac Fibroblasts Via Activation of Protease-Activated Receptor-1. Cells.

[38] Cardin S., Li D., Thorin-Trescases N., Leung T.K., Thorin E., Nattel S. (2003). Evolution of the Atrial Fibrillation Substrate in Experimental Congestive Heart Failure: Angiotensin-Dependent and -Independent Pathways. Cardiovasc. Res..

[39] Goette A., Staack T., Rocken C., Arndt M., Geller J.C., Huth C., Ansorge S., Klein H.U., Lendeckel U. (2000). Increased Expression of Extracellular Signal-Regulated Kinase and Angiotensin-Converting Enzyme in Human Atria during Atrial Fibrillation. J. Am. Coll. Cardiol..

[40] Sabri A., Short J., Guo J., Steinberg S.F. (2002). Protease-Activated Receptor-1-Mediated DNA Synthesis in Cardiac Fibroblast Is Via Epidermal Growth Factor Receptor Transactivation: Distinct Par-1 Signaling Pathways in Cardiac Fibroblasts and Cardiomyocytes. Circ. Res..

[41] Climent A.M., Guillem M.S., Fuentes L., Lee P., Bollensdorff C., Fernandez-Santos M.E., Suarez-Sancho S., Sanz-Ruiz R., Sanchez P.L., Atienza F. (2015). Role of Atrial Tissue Remodeling on Rotor Dynamics: An in Vitro Study. Am. J. Physiol. Heart Circ. Physiol..

[42] van Gorp P.R.R., Trines S.A., Pijnappels D.A., de Vries A.A.F. (2020). Multicellular in Vitro Models of Cardiac Arrhythmias: Focus on Atrial Fibrillation. Front. Cardiovasc. Med..

[43] Sartiani L., Bochet P., Cerbai E., Mugelli A., Fischmeister R. (2002). Functional Expression of the Hyperpolarization-Activated, Non-Selective Cation Current I(F) in Immortalized Hl-1 Cardiomyocytes. J. Physiol..

[44] Dias P., Desplantez T., El-Harasis M.A., Chowdhury R.A., Ullrich N.D., Cabestrero de Diego A., Peters N.S., Severs N.J., MacLeod K.T., Dupont E. (2014). Characterisation of Connexin Expression and Electrophysiological Properties in Stable Clones of the Hl-1 Myocyte Cell Line. PLoS ONE.

[45] Monge C., Beraud N., Tepp K., Pelloux S., Chahboun S., Kaambre T., Kadaja L., Roosimaa M., Piirsoo A., Tourneur Y. (2009). Comparative Analysis of the Bioenergetics of Adult Cardiomyocytes and Nonbeating Hl-1 Cells: Respiratory Chain Activities, Glycolytic Enzyme Profiles, and Metabolic Fluxes. Can. J. Physiol. Pharmacol..

[46] Kuznetsov A.V., Javadov S., Sickinger S., Frotschnig S., Grimm M. (2015). H9c2 and Hl-1 Cells Demonstrate Distinct Features of Energy Metabolism, Mitochondrial Function and Sensitivity to Hypoxia-Reoxygenation. Biochim. Biophys. Acta.

[47] Derian C.K., Santulli R.J., Tomko K.A., Haertlein B.J., Andrade-Gordon P. (1995). Species Differences in Platelet Responses to Thrombin and Sfllrn. Receptor-Mediated Calcium Mobilization and Aggregation, and Regulation by Protein Kinases. Thromb. Res..

[48] Nakajima H., Nakajima H.O., Salcher O., Dittie A.S., Dembowsky K., Jing S., Field L.J. (2000). Atrial but Not Ventricular Fibrosis in Mice Expressing a Mutant Transforming Growth Factor-Beta(1) Transgene in the Heart. Circ. Res..

[49] Li F., Wang X., Capasso J.M., Gerdes A.M. (1996). Rapid Transition of Cardiac Myocytes from Hyperplasia to Hypertrophy During Postnatal Development. J. Mol. Cell. Cardiol..

[50] Soonpaa M.H., Kim K.K., Pajak L., Franklin M., Field L.J. (1996). Cardiomyocyte DNA Synthesis and Binucleation During Murine Development. Am. J. Physiol..

[51] Onódi Z., Visnovitz T., Kiss B., Hambalkó S., Koncz A., Ágg B., Váradi B., Tóth V., Nagy R.N., Gergely T.G. (2022). Systematic Transcriptomic and Phenotypic Characterization of Human and Murine Cardiac Myocyte Cell Lines and Primary Cardiomyocytes Reveals Serious Limitations and Low Resemblances to Adult Cardiac Phenotype. J. Mol. Cell. Cardiol..

[52] Trejo J. (2003). Protease-Activated Receptors: New Concepts in Regulation of G Protein-Coupled Receptor Signaling and Trafficking. J. Pharmacol. Exp. Ther..

[53] Rauch B.H., Millette E., Kenagy R.D., Daum G., Clowes A.W. (2004). Thrombin- and Factor Xa-Induced DNA Synthesis Is Mediated by Transactivation of Fibroblast Growth Factor Receptor-1 in Human Vascular Smooth Muscle Cells. Circ. Res..

[54] Friebel J., Weithauser A., Witkowski M., Rauch B.H., Savvatis K., Dorner A., Tabaraie T., Kasner M., Moos V., Bosel D. (2019). Protease-Activated Receptor 2 Deficiency Mediates Cardiac Fibrosis and Diastolic Dysfunction. Eur. Heart J..

[55] Albitz R., Droogmans G., Nilius B., Casteels R. (1992). Thrombin Stimulates L-Type Calcium Channels of Guinea Pig Cardiomyocytes in Cell-Attached Patches but Not after Intracellular Dialysis. Cell Calcium.

[56] Lin C., Rezaee F., Waasdorp M., Shi K., van der Poll T., Borensztajn K., Spek C.A. (2015). Protease Activated Receptor-1 Regulates Macrophage-Mediated Cellular Senescence: A Risk for Idiopathic Pulmonary Fibrosis. Oncotarget.

[57] Borensztajn K., Aberson H., Peppelenbosch M.P., Spek C.A. (2009). Fxa-Induced Intracellular Signaling Links Coagulation to Neoangiogenesis: Potential Implications for Fibrosis. Biochim. Biophys. Acta.

[58] Dorn L.E., Petrosino J.M., Wright P., Accornero F. (2018). Ctgf/Ccn2 Is an Autocrine Regulator of Cardiac Fibrosis. J. Mol. Cell. Cardiol..

[59] Salvarani N., Maguy A., De Simone S.A., Miragoli M., Jousset F., Rohr S. (2017). Tgf-Β1(Transforming Growth Factor-Β1) Plays a Pivotal Role in Cardiac Myofibroblast Arrhythmogenicity. Circ. Arrhythmia Electrophysiol..

[60] Grand T., Salvarani N., Jousset F., Rohr S. (2014). Aggravation of Cardiac Myofibroblast Arrhythmogeneicity by Mechanical Stress. Cardiovasc. Res..

[61] Jiang T., Danilo P., Steinberg S.F. (1998). The Thrombin Receptor Elevates Intracellular Calcium in Adult Rat Ventricular Myocytes. J. Mol. Cell. Cardiol..

[62] Macfarlane S.R., Sloss C.M., Cameron P., Kanke T., McKenzie R.C., Plevin R. (2005). The Role of Intracellular Ca^2+^ in the Regulation of Proteinase-Activated Receptor-2 Mediated Nuclear Factor Kappa B Signalling in Keratinocytes. Br. J. Pharmacol..

[63] Di Scipio R.G., Hermodson M.A., Yates S.G., Davie E.W. (1977). A Comparison of Human Prothrombin, Factor Ix (Christmas Factor), Factor X (Stuart Factor), and Protein S. Biochemistry.

[64] Lawson J.H., Kalafatis M., Stram S., Mann K.G. (1994). A Model for the Tissue Factor Pathway to Thrombin. I. An Empirical Study. J. Biol. Chem..

[65] Riewald M., Kravchenko V.V., Petrovan R.J., O’Brien P.J., Brass L.F., Ulevitch R.J., Ruf W. (2001). Gene Induction by Coagulation Factor Xa Is Mediated by Activation of Protease-Activated Receptor 1. Blood.

[66] Riewald M., Ruf W. (2001). Mechanistic Coupling of Protease Signaling and Initiation of Coagulation by Tissue Factor. Proc. Natl. Acad. Sci. USA.

